# Long-term safety and survival outcomes from the Scandinavian Breast Group 2004-1 randomized phase II trial of tailored dose-dense adjuvant chemotherapy for early breast cancer

**DOI:** 10.1007/s10549-017-4599-4

**Published:** 2017-11-30

**Authors:** Alexios Matikas, Sara Margolin, Mats Hellström, Hemming Johansson, Nils-Olof Bengtsson, Lena Karlsson, Per Edlund, Per Karlsson, Elisabet Lidbrink, Barbro Linderholm, Henrik Lindman, Per Malmstrom, Kenneth Villman, Theodoros Foukakis, Jonas Bergh

**Affiliations:** 1 Department of Oncology, Radiumhemmet, Karolinska University Hospital, Stockholm, Sweden; 2 Department of Oncology, Stockholm South General Hospital, Stockholm, Sweden; 3 Norrland University Hospital, Umeå, Sweden; 4 County Hospital, Sundsvall, Sweden; 5 Gävle Hospital, Gävle, Sweden; 6 Department of Oncology, Institute of Clinical Sciences, Sahlgrenska Academy, Sahlgrenska University Hospital, University of Gothenburg, Gothenburg, Sweden; 7 Department of Oncology, Uppsala University Hospital, Uppsala, Sweden; 8 Department of Oncology and Radiation Physics, Skåne University Hospital, Lund, Sweden; 9 Örebro University Hospital, Örebro, Sweden

**Keywords:** Adjuvant chemotherapy, Breast cancer, Dose-dense, Randomized, Tailored dose

## Abstract

**Purpose:**

Although adjuvant polychemotherapy improves outcomes for early breast cancer, the significant variability in terms of pharmacokinetics results in differences in efficacy and both short and long-term toxicities. Retrospective studies support the use of dose tailoring according to the hematologic nadirs.

**Methods:**

The SBG 2004-1 trial was a randomized feasibility phase II study which assessed tailored dose-dense epirubicin and cyclophosphamide (EC) followed by docetaxel (T) (group A), the same regimen with fixed doses (group B) and the TAC regimen (group C). Women aged 18–65 years, ECOG PS 0-1 with at least one positive axillary lymph node were randomized 1:1:1. The primary endpoint of the study was the safety and feasibility of the treatment. Toxicity was graded according to CTC-AE version 3.0. The design and short-term toxicity have been previously published. Here, we report safety and efficacy data after 10 years of follow-up.

**Results:**

A total of 124 patients were included in the study. After a median follow-up of 10.3 years, the probability for 10-year survival was 78.5, 75.1, and 63.4% and for relapse free survival 64.1, 71.0, and 59.5% for groups A, B, and C, respectively. There were no cases of clinically diagnosed cardiotoxicity or hematologic malignancies. No patient was lost to follow-up.

**Conclusions:**

In this randomized phase II trial, tailored dose adjuvant chemotherapy was feasible, without an increased risk for long-term adverse events after a median follow-up of 10 years.

## Introduction

The administration of adjuvant polychemotherapy (ACT) after surgery for early and locally advanced breast cancer (BC) has been consistently shown to decrease both breast cancer-specific and overall mortality [1]. Multiple efforts have been undertaken in order to improve the concurrent or sequential administration of an anthracycline and taxane, including either the escalation of administered doses (dose intense chemotherapy) or the administration of conventional doses in shorter time intervals (dose-dense chemotherapy, DD-CT). Dose escalation above a certain threshold without patient selection, including high-dose therapy and autologous bone marrow support, has generally failed to prolong survival [2–5]. On the other hand, several but not all trials have reported improved outcomes with the use of DD-CT [6–10]. However, attempts to identify which patients benefit the most from such an approach have been largely unsuccessful. Although a trial-level meta-analysis concluded that only patients with estrogen receptor (ER) negative disease had a significantly improved overall survival benefit from DD-CT [11], the findings of individual randomized trials [7, 10] reveal a significant benefit in DFS for both ER-positive and ER-negative disease. Furthermore, the Early Breast Cancer Trialists’ Collaborative Group (EBCTCG) meta-analysis reported similar proportional reductions in risk of death induced by ACT regardless of ER status; the same relative reduction was also found to be independent of age, tumor size, axillary lymph node status, and use of tamoxifen [1].

Utilizing hematologic nadirs as a guidance for subsequent dosing represents an attempt to circumvent the inherent inability of body surface area to account for the significant inter-patient variabilities in the pharmacokinetic and pharmacodynamic properties of chemotherapeutic agents [12]. Supporting the notion of tailored dose chemotherapy (td-CT) are the results of five retrospective analyses of approximately 2000 patients, which reported an association between clinical outcomes after ACT for BC and the depth of neutropenia experienced during treatment [13–17].

Based on these observations, the Scandinavian Breast Group (SBG) 2004-1 trial was a randomized feasibility/phase II study which evaluated the safety of a tailored, dose-dense regimen of epirubicin and cyclophosphamide (EC) followed by docetaxel (T), compared to the same regimen with fixed doses and the combination of docetaxel, doxorubicin, and cyclophosphamide (TAC), in patients with node-positive BC. The trial completed enrollment in 2006, and short-term safety results have been previously published [18]. Here we present updated survival and safety outcomes after a median follow-up of over 10 years.

## Patients and methods

### Study design

The SBG 2004-1 was a prospective, randomized, multicenter phase II trial which compared three different regimens as adjuvant treatment for completely resected BC. The trial’s design has been previously presented in detail [18]. The study was conducted in 10 collaborative centers of the SBG in Sweden. The study protocol was approved by the ethics committee at the Karolinska Institute and by the Swedish Medical Product Agency. Written informed consent was required from all patients prior to enrollment.

### Patients

Women aged 18–65 years with histologically confirmed, surgically resected early invasive breast adenocarcinoma, and with an Eastern Cooperative Oncology Group (ECOG) performance status (PS) of 0 or 1 were eligible for this study. All enrolled patients had undergone primary surgery with tumor-free margins. On pathologic examination, macrometastatic disease to at least one axillary lymph node was mandatory for patients with ER negative and to four lymph nodes for ER-positive disease. Key exclusion criteria included the presence of distant metastases, impaired baseline cardiac, liver or hematologic function, and the presence of a second non-breast malignancy.

### Treatment Plan

Patients were randomly assigned to one of three treatment groups: tailored epirubicin and cyclophosphamide every 2 weeks followed by tailored docetaxel every 2 weeks, each for 4 cycles (arm A, tdEC → tdT) fixed dose epirubicin and cyclophosphamide every 2 weeks followed by fixed dose docetaxel every 2 weeks, each administered for 4 cycles [arm B, E_90_C_600_ → T_75_ (mg/m^2^)]; or six cycles of the TAC regimen [arm C, T_75_A_50_C_500_ (mg/m^2^)] administered every 3 weeks (Table 1). The dosing considerations and guidelines regarding the use of supportive medications in this trial (antiemetics, myeloid growth factors and ciprofloxacin) have been previously described in detail [18]. Following the completion of chemotherapy, all enrolled patients received standard of care adjuvant radiotherapy, trastuzumab, and endocrine therapy as indicated according to contemporary national and regional clinical practice guidelines.

**Table 1:** Administered treatment

	Dose step	Epirubicin (E, mg/m^2^)	Cyclophosphamide (C, mg/m^2^)	Docetaxel (T, mg/m^2^)	Treatment schedule
Arm A	− 3	38	450	–	4 × EC → 4 × T, every 2 weeks. Start at dose level 1 and adapt doses according to hematologic toxicity
	− 2	60	600	–	
	− 1	75	600	60	
	1	90	600	75	
	2	105	900	85	
	3	120	1200	100	
Arm B	–	90	600	75	4 × EC → 4 × T, every 2 weeks
Arm C	–	50 (doxorubicin, A)	500	75	6 × TAC, every 3 weeks

### Outcomes

The primary endpoint of the study was the safety and feasibility of the administered treatment. The secondary endpoint of the study was to evaluate the dose intensity of the three regimens. Toxicity was assessed at baseline and following each treatment cycle and grading used the Common Terminology Criteria for Adverse Events of the National Cancer Institute version 3.0.

### Statistical considerations

The primary endpoint of the study was to evaluate the feasibility and safety of the three treatment alternatives and to choose either arm B or C for the continuation Phase III part as comparators to the tailored dose-dense arm A. The primary goal was to estimate the proportion of patients receiving planned treatment with less or equal than 20% delay or the proportion of patients that require hospitalization due to side effects of the treatment. As a result, it was estimated that 40 patients were needed in each treatment arm. Continuous variables were summarized with descriptive statistics (n, mean, standard deviation, range, and median). Since efficacy endpoints were not predefined for the feasibility phase II part of the study, for the scope of this analysis, we performed an exploratory efficacy analysis using the endpoints of the continuation phase III part. Breast cancer recurrence-free survival (RFS) was defined as time from randomization to the first of the events: local, regional, or distant breast cancer recurrence and death due to breast cancer or last date of follow-up if no event has occurred. Event-free survival (EFS) was defined as the time from randomization to the first of the events: breast cancer recurrence (any type), contra-lateral breast cancer, other malignancy, and any cause of death. Overall survival (OS) was measured from the time of randomization until the date of death from any cause. RFS, EFS, and OS for all patients were estimated using the Kaplan–Meier analysis and the comparisons were computed with the log-rank test. Since the outcome analysis was not predefined, no formal comparisons were performed. All statistical tests were two-sided, and p-values < 0.05 were considered statistically significant. All statistical analyses were performed using SPSS v.22.0 (IBM Corp., Armonk, NY, USA).

## Results

### Patient characteristics

The clinical and demographic characteristics of the 124 patients treated in this study have been previously described in detail [18]. All patients except for one who withdrew consent right after randomization to treatment group C received at least one dose of adjuvant chemotherapy. Post-chemotherapy adjuvant endocrine therapy was balanced between the three treatment groups, with 25/42 (59.5%) patients in treatment group A, 28/42 (66.6%) in group B, and 23/40 (57.5%) in group C.

### Outcomes

**Fig. 1:**
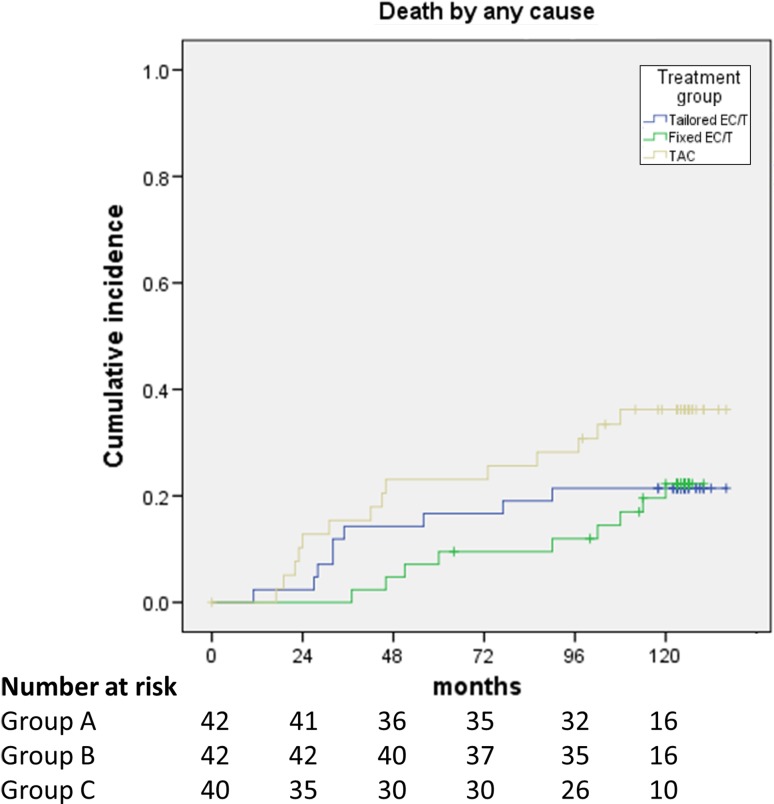
Cumulative incidence of death by any cause. EC: epirubicin and cyclophosphamide; T: docetaxel; TAC: docetaxel, doxorubicin, and cyclophosphamide

After a median follow-up of 10.3 years, 32 patients had died: 9 among those treated in treatment group A, 9 in group B, and 14 in group C. Although the study was not designed or powered to perform formal comparisons, all three regimens were associated with good long-term survival, in light of the high-risk population. In the exploratory survival analysis of the intention-to-treat population, the median OS could not be estimated in neither of the three groups. The cumulative probability for survival at five and ten years was, for treatment A 80.9% and 78.5%, for treatment B 90.4% and 75.1%, and for treatment C 74.4% and 63.4%, respectively (Fig. 1).

**Fig. 2:**
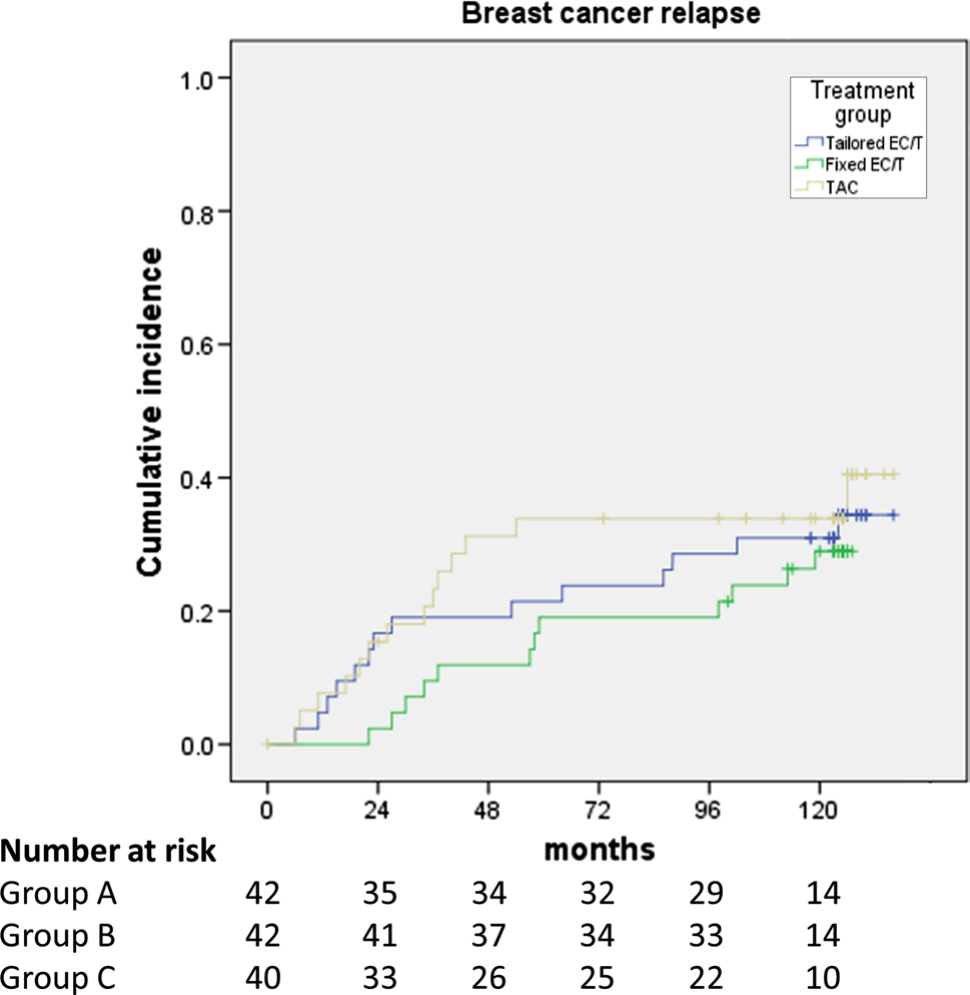
Cumulative incidence of breast cancer relapse. EC: epirubicin and cyclophosphamide; T: docetaxel; TAC: docetaxel, doxorubicin, and cyclophosphamide

**Fig. 3:**
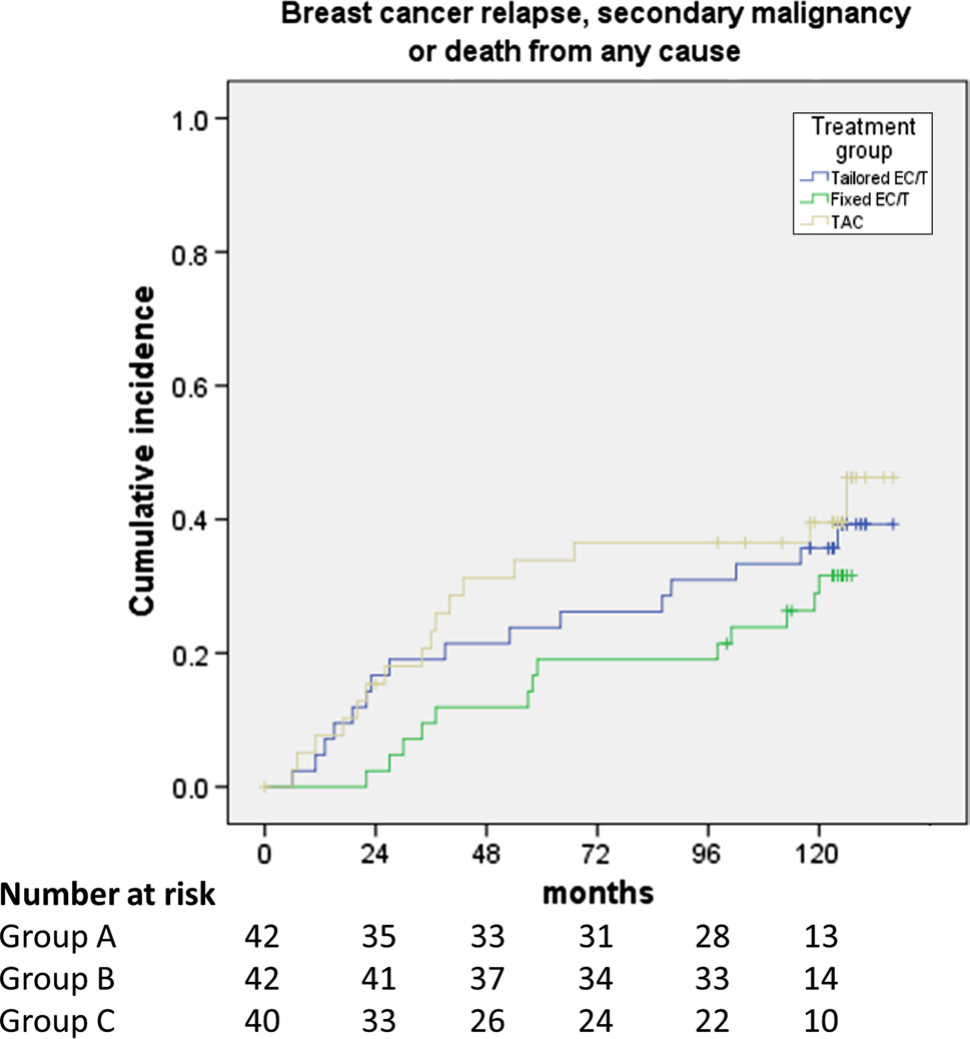
Cumulative incidence of breast cancer relapse, secondary malignancy, or death by any cause. EC: epirubicin and cyclophosphamide; T: docetaxel; TAC: docetaxel, doxorubicin, and cyclophosphamide

At the time of data cutoff, 40 patients had relapsed: 14 patients treated with regimen A, 12 with regimen B, and 14 with regimen C. The median RFS could not be estimated in neither of the three groups. The cumulative probability for relapse-free survival at five and 10 years was 76.2 and 64.1% for treatment A, 80.9 and 71.0% for treatment B, and 66.1 and 59.5% for treatment C, respectively (Fig. 2). The respective probabilities for EFS at five and 10 years were 73.8% and 59.2% for treatment A, 80.9 and 66.0% for treatment B, and 63.5 and 54.0% for treatment C, respectively (Fig. 3). The differences in terms of OS, RFS, and EFS between the three treatment arms were not statistically significant.

### Long-term safety

The short-term safety considerations of this trial have been previously described [18]. Using a follow-up scheme based on triggering further evaluation depending on symptom reporting and physical examination, no significant long-term adverse events were documented. With a median follow-up of 10 years, there were no cases of hematological malignancies (acute myeloid leukemias or myelodysplastic syndromes, AML/MDS). Four solid malignancies were reported, two in patients from group A and two from group C, all considered not related to the study treatments: one case of gastric cancer, a squamous cell cancer of the cervix, a jejunal gastrointestinal stromal tumor, and a cutaneous melanoma. The median latency period between patient registration and diagnosis of the secondary malignancy was 91.6 months.

## Discussion

After a median follow-up of 10 years, tailored ACT with EC followed by T was associated with excellent long-term safety, since there were no documented cases of hematologic malignancies or clinically diagnosed cardiotoxicity. An important long-term adverse event of ACT for BC is the development of marrow neoplasias, with an approximately seven-fold increase in cumulative risk at 10 years reported in a large retrospective analysis [19]. Both anthracyclines and alkylating agents, two commonly used drug classes in ACT regimens, have been linked with secondary acute myeloid leukemia and/or myelodysplastic syndrome (AML/MDS), which frequently exhibit distinct karyotypic and molecular abnormalities and confer a poor prognosis [20–22]. The risk is higher with larger cumulative doses of anthracyclines and cyclophosphamide, regardless of dose tailoring [23–25]. In contrast, both in a study of 6 courses of neoadjuvant tailored FEC [26] and in the present study after a longer follow-up period, no cases of AML/MDS were reported. Although one could argue that the small number of patients that received the experimental treatment in SBG 2004-1 may have masked such an association, the safety outcomes reported from the larger PANTHER study do not support this hypothesis [27], since there were three cases of AML/MDS in the experimental group compared with two cases in the standard treatment group at a median follow-up of 5.3 years, which should be sufficient for most topoisomerase II-related leukemias [25]. Rather, the low incidence of AML/MDS in these two studies probably reflects the lower cumulative doses of cyclophosphamide administered compared to trials that have explored dose intense and dose-dense ACT [10], but also likely due the relatively low cumulative median dose of epirubicin of 406 mg/m^2^ in the Panther study.

The tailored dose regimen resulted in numerically superior long-term outcomes compared to six cycles of TAC, a regimen that has been found to be equivalent to the dose-dense sequential administration of doxorubicin and paclitaxel [8]. Although this feasibility trial was not powered for such a comparison, the continuation phase III part of this study, denoted the PANTHER, confirmed the efficacy of the tailored approach. In PANTHER, the same regimen as the one used in SBG 2004-1 resulted in borderline favorable trends in terms of both RFS and OS when compared to 5-fluorouracil combined with EC (FE_100_C) followed by T_100_ as in the PACS 01 trial [28], while the improvement in event-free survival was statistically significant. All the predefined endpoints had hazard ratios around 0.8 (0.77–0.83) [27].

The efficacy of the experimental tailored regimen is based on three premises: Firstly, the Norton–Simon hypothesis states that the rate of cancer cell death is directly proportional to the tumor growth rate at the time of its administration. As tumor cell growth increases between chemotherapy cycles according to Gompertzian kinetics, reducing the interval results in more effective suppression of tumor regrowth and faster cell-kill [29]. Secondly, escalating doses of certain chemotherapeutics that exhibit linear pharmacokinetics may overcome resistance and eradicate clones that exhibit low sensitivity to treatment [30]. Finally, the new onset of agent-specific adverse events has been repeatedly linked with improved outcomes after treatment for several solid malignancies [31–35]. Conceivably, these toxicities can be used as readily apparent surrogate markers for the metabolism of certain agents; thus, tailoring doses based on their appearance and severity potentially counteracts the risks of both undertreatment which may impair outcomes and overtreatment which exposes patients to unnecessary toxicity.

In conclusion, updated results from the SBG 2004-1 trial indicate that td-CT is feasible and safe, without increased long-term toxicities. The safety data from this trial in combination with the safety and efficacy data from the PANTHER study imply that dose tailoring may be the next step in the evolution of ACT strategies in BC.
